# Numerical and Experimental Analysis of Buckling and Post-Buckling Behaviour of TWCFS Lipped Channel Section Members Subjected to Eccentric Compression

**DOI:** 10.3390/ma17122874

**Published:** 2024-06-12

**Authors:** Jacek Jankowski, Maria Kotełko, Viorel Ungureanu

**Affiliations:** 1 Faculty of Mechanical Engineering, Łódź University of Technology, 90-537 Łódź, Poland; maria.kotelko@p.lodz.pl; 2 Department of Steel Structures and Structural Mechanics, Civil Engineering Faculty, Politehnica University Timisoara, 300224 Timisoara, Romania; viorel.ungureanu@upt.ro; 3 Laboratory of Steel Structures, CCTFA, Romanian Academy—Timisoara Branch, 300223 Timisoara, Romania; 4 Technical Science Academy of Romania, 010413 Bucharest, Romania

**Keywords:** buckling, eccentricity, experiment, finite element method, finite difference method, lipped channel cross-section member, post-buckling, thin-walled structure

## Abstract

The paper presents a static analysis of the buckling and post-buckling state of thin-walled cold-formed steel (TWCFS) lipped channel section beam-columns subjected to eccentric compression. Eccentricity is taken into consideration relative to both major and minor principal axes. An analytical–numerical solution to the buckling and post-buckling problems is described. The solution is based on the theory of thin plates. Equations of equilibrium of section walls are derived from the principle of stationary energy. Then, to solve the problem, the finite difference (FDM) and Newton–Raphson methods are applied. Linear (buckling) and nonlinear (post-buckling) analyses are performed. As a result, pre- and post-buckling equilibrium paths are determined. Comparisons of the obtained numerical results, FE simulation results, and experimental test results are carried out and presented in comparative load-shortening diagrams. Additionally, a comparison of the buckling forces and buckling modes obtained from theoretical analysis and experiments is presented.

## 1. Introduction

Thin-walled cold-formed steel (TWCFS) structures are widely used because of their low cost, high performance, and relatively uncomplicated production. These structures have high mechanical parameters, especially load-carrying capacity. However, the capacities of these members are reduced due to local, distortional, or global buckling.

The phenomenon of buckling of TWCFS members subjected to pure bending or uniform compression is solved using EN 1993-1-3 [1]. In that case, it gives a good prediction of the buckling and ultimate strength, but in the case of eccentric compression, the standards give less convergence in comparison with the experimental data.

Relatively few publications report both theoretical and experimental results concerning buckling and ultimate strength of thin-walled cold-formed steel members of mono-symmetric cross sections under eccentric compression, especially concerning the major axis bending.

He et al. [2] investigated built-up sections (back-to-back) with V- or Σ-shaped web stiffeners subjected to concentric and eccentric compression using the finite element method (FEM), validated with experimental results.

Zhao et al. [3] presented the results of strength predictions based on the direct strength method (DSM) compared to the results of an experimental test performed on members of a perforated TWCFS channel section subjected to eccentric compression.

In [4], an eccentrically loaded TWCFS beam-column of a lipped and sigma channel section was investigated. Axial compression and biaxial bending moments cases were tested. Moreover, analytical and finite element method (FEM) analyses were carried out. For the numerical simulations, ABAQUS software was used.

Guo and Fukumoto [5] solved the post-buckling problem and numerically calculated the load-carrying capacity of thin-walled, cold-formed, and welded columns loaded eccentrically. The solution of this problem was based on the finite strip method. The researchers also conducted experimental tests. The experimental data were compared with the numerical data.

Kotełko et al. [6] took into account the possibility of using local plastic mechanisms to calculate the ultimate strength of short TWCFS members of a lipped channel section subjected to eccentric compression. In this paper, a database of local plastic failure mechanisms was created for members subjected to eccentric compression on the minor axis.

Borkowski et al. [7] presented the results of experiments dealing with the post-failure behaviour of members of a TWCFS lipped channel section subjected to eccentric compression around the minor axis for various eccentricity values, considering positive or negative values. Furthermore, the data from the FE simulations were compared with the experimental data.

Ungureanu et al. [8,9] investigated experimentally the influence of eccentricity on the structural behaviour (buckling, ultimate strength, and post-ultimate behaviour) of stub columns made of TWCFS lipped channel sections subjected to eccentric compression about both the minor and the major axes, followed by validation and calibration of FE models and identification and calibration of failure mechanisms developed in the post-ultimate stage.

Rajkannu and Arul Jayachandran [10] presented a design of TWCFS beam-columns using the direct strength method (DSM) and compared this with experimental results. During the experiment, a specimen was subjected to pure bending using moments about both the major and the minor axes. The authors tried to change the approach to improve their results from the strength prediction calculations. The results were closer to the experimental data.

Peiris and Mahendran [11] analysed short and intermediate TWCFS members subjected to eccentric compression. They obtained load vs. shortening diagrams. In addition, the failure modes were analysed. The finite element method was used and developed. This was validated using the results of the experiment. The results showed that the DSM to predict load-carrying capacity is less accurate.

Zhang et al. [12] dealt with the influence of combined compression and bending moment about the major axis on the cross-section behaviour of press-braked stainless steel channel sections. The authors developed a tool to solve the problem, including initial local shape imperfections. First, the finite element method was developed and validated with the test results.

Liang et al. [13] investigated the local cross-section behaviour of stainless steel channel section members subjected to combined axial compression and bending moment around the minor axis. A finite element simulation study on stainless steel channel sections under combined compression and bending moment around the minor axis is presented in [14]. The main aim of this work was to show that standards give less accurate results of the calculation of the ultimate load capacity, but the modified FEM gives results close to the experimental data.

Another study by Liang et al. [15] presents an experimental and numerical analysis of the behaviour of laser-welded stainless steel channel sections subjected to combined compression and bending moment about the major axis. In the experimental investigation, two laser-welded austenitic stainless steel plain channel sections were considered, and for each channel section, four eccentricities were analysed. Finite element models were validated with experimental data.

Craveiro et al. [16] investigated experimentally, numerically, and analytically the buckling behaviour of closed built-up cold-formed steel columns with four different cross-sectional shapes (i.e., back-to back types of structures) under axial compression. They observed that for the closed built-up cold-formed steel short columns, they failed by local buckling. They also observed the outward movement of the flanges of the plain channels between the fasteners. They achieved good agreement of the experimental data with the rest of the results (from FEM and standards).

While many works present the results of buckling and post-buckling analysis obtained using the finite element or finite strip method, there are very few publications applying the finite difference method (FDM) to a solution of those problems taking into consideration thin plate theory. Among them, a paper worthy of mention was by An et al. [17], who performed bending and buckling analysis of irregular plates made of functionally graded graphene origami metamaterial using the finite difference method (FDM). They investigated metamaterial irregular plates (octagonal star plates, cordiform plates, a quarter of annular plates, and a quarter of annular plates with a hole) under different boundary conditions. The analysis was based on higher-order shear deformation theory.

The above literature review shows some gaps in research on buckling and post-buckling behaviour of short TWCFS open section members subject to combined compression and bending, particularly eccentric compression. Therefore, the objective of the present study is the analysis of buckling and post-buckling behaviour, in the context of the applicability of this analysis to the estimation of the load-carrying capacity of the members investigated, by the compilation of equilibrium paths obtained from buckling and post-buckling with post-ultimate paths obtained using the plastic mechanism curve [8]. To perform this analysis, the finite difference (FD) and Newton–Raphson methods were adopted to solve the buckling and post-buckling problem of TWCFS lipped channel section members subject to eccentric compression about both the major and minor axes. A comparison of the numerical (linear and nonlinear) analysis results and the experimental results was performed. The theoretical semi-analytical approach presented can be used to predict behaviour of TWCFS members before reaching an ultimate load and post-ultimate state. As mentioned above, the post-buckling equilibrium path obtained from the analysis, together with the post-ultimate curve, provides an upper-bound estimate of the load-carrying capacity. This estimation can be incorporated into the standard procedure.

## 2. Basics of the Numerical Solution of the Problem

**Figure 1:**
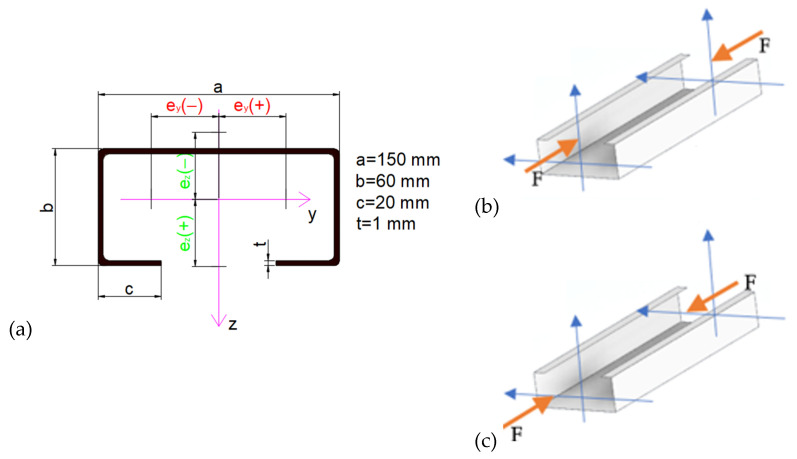
(**a**) Cross section of the analysed member with dimensions (length of member L = 450 mm), global coordinate system and eccentricity about principal (minor and major) axes [9], (**b**,**c**) member subjected to eccentric compression about minor and major axes correspondingly.

The general dimensions of the investigated TWCFS member are shown in Figure 1. The structure consists of thin plates (walls).

Static equilibrium of the plates (web, flanges, and lips) is described using equilibrium equations and equations of continuity between the two closest plates. The equilibrium equations were obtained using variation in the potential energy of a single plate.

### 2.1. General Formulations

Components (1) of the strain tensor for the membrane and bending state of the single plate [14] for the eccentricity (e_y_) measured relative to the major axis (z) are as follows:

(1a) $$\begin{array}{l} \varepsilon_{x}=u_{,x}+{0.5w}_{,x}^{2}-z\cdot w_{,\mathrm{xx}}\\ \varepsilon_{y}=v_{,y}+0.5{(v}_{,y}^{2}+w_{,y}^{2})-{z\cdot w}_{,\mathrm{yy}}\\ \gamma_{\mathrm{xy}}=u_{,y}+v_{,x}+w_{,x}w_{,y}-2\cdot z{\cdot w}_{,\mathrm{xy}} \end{array}$$

and for the eccentricity (e_z_) measured relative to the (y) axis (minor axis):

(1b) $$\begin{array}{l} \varepsilon_{x}=u_{,x}+{0.5{(v}_{,x}^{2}+w}_{,x}^{2})-z\cdot w_{,\mathrm{xx}}\\ \varepsilon_{y}=v_{,y}+0.5\;w_{,y}^{2}-{z\cdot w}_{,\mathrm{yy}}\\ \gamma_{\mathrm{xy}}=u_{,y}+v_{,x}+w_{,x}w_{,y}-2\cdot z{\cdot w}_{,\mathrm{xy}} \end{array}$$

where u, v, and w are displacements of the middle surface of a single plate and (z) is the coordinate measured from the middle surface. Constitutive equations in a well-known form for an isotropic material are:

(2) $$\begin{array}{l} \sigma_{x}=\frac{E}{1-\nu^{2}}\left(\varepsilon_{x}+\nu \varepsilon_{y}\right)\\ \sigma_{y}=\frac{E}{1-\nu^{2}}\left(\varepsilon_{y}+\nu \varepsilon_{x}\right)\\ \tau_{\mathrm{xy}}=G\gamma_{\mathrm{xy}} \end{array}$$

where G = E/(2(1 + v)), E is Young’s modulus and ν is Poisson’s ratio. Internal forces and moments are defined as follows:

(3) $$\begin{array}{l} N_{x}=\int_{-\frac{t}{2}}^{\frac{t}{2}}\sigma_{x}\mathrm{dz}=\frac{E\cdot t}{1-\nu^{2}}\left(\varepsilon_{x}+\nu \varepsilon_{y}\right)\\ N_{y}=\int_{-\frac{t}{2}}^{\frac{t}{2}}\sigma_{y}\mathrm{dz}=\frac{E\cdot t}{1-\nu^{2}}(\varepsilon_{y}+\nu \varepsilon_{x})\\ N_{\mathrm{xy}}=\int_{-\frac{t}{2}}^{\frac{t}{2}}\tau_{\mathrm{xy}}\mathrm{dz}=\mathrm{Gt}\gamma_{\mathrm{xy}}\\ M_{x}=\int_{-\frac{t}{2}}^{\frac{t}{2}}\sigma_{x}\mathrm{zdz}=-D(w_{,\mathrm{xx}}+\nu {\cdot w}_{,\mathrm{yy}})\\ M_{y}=\int_{-\frac{t}{2}}^{\frac{t}{2}}\sigma_{y}\mathrm{zdz}=-D(w_{,\mathrm{yy}}+\nu {\cdot w}_{,\mathrm{xx}})\\ M_{\mathrm{xy}}=\int_{-\frac{t}{2}}^{\frac{t}{2}}\tau_{\mathrm{xy}}\mathrm{zdz}=D(1-\nu ){\cdot w}_{,\mathrm{xy}} \end{array}$$

where t is the thickness of the plate, N_x_, N_y_, and N_xy_ [N/m] the internal forces, M_x_, M_y_ [N] the bending moments, and M_xy_ [N] the torque on the middle surface of the plate.

Potential energy Π is formulated as [18]:

(4) $$\Pi \;=\int \left(\sigma_{x}\varepsilon_{x}+\sigma_{y}\varepsilon_{y}+\tau_{\mathrm{xy}}\gamma_{\mathrm{xy}}\right)\mathrm{dV}-\int_{0}^{b}tp^{0}\mathrm{udy}\left|\begin{array}{c} x=L\\ x=0 \end{array}\right.$$

This is the total energy of the single thin plate, where b is the width of plate, $p^{0}$ is the external load before buckling on the middle surface of the plate, and V is the volume in potential energy (4), which takes the form [18]:

(5) $$\delta \Pi \;=\int \left(\sigma_{x}{\delta \varepsilon }_{x}+\sigma_{y}{\delta \varepsilon }_{y}+\tau_{\mathrm{xy}}{\delta \gamma }_{\mathrm{xy}}\right)\mathrm{dV}\;-\int_{0}^{b}tp^{0}\delta \mathrm{udy}\left|\begin{array}{c} x=L\\ x=0 \end{array}\right.=0$$

and (according to the principle of stationary energy) is equal to zero.

From Equation (5), after grouping terms standing by corresponding variations of displacements, three equilibrium equations are obtained as follows (major axis):

(6a) $$\begin{array}{l} N_{x,x}+N_{\mathrm{xy},y}=\;0\\ N_{y,y}+N_{\mathrm{xy},x}+{\left(N_{y}v_{,y}\right)}_{,y}=\;0\\ M_{x,\mathrm{xx}}+M_{y,\mathrm{yy}}+{2M}_{\mathrm{xy},\mathrm{xy}}+{\left(N_{x}w_{,x}\right)}_{,x}+{\left(N_{y}w_{,y}\right)}_{,y}+{\left(N_{\mathrm{xy}}w_{,x}\right)}_{,y}+{\left(N_{\mathrm{xy}}w_{,y}\right)}_{,x}=\;0 \end{array}$$

and for the minor axis:

(6b) $$\begin{array}{l} N_{x,x}+N_{\mathrm{xy},y}=\;0\\ N_{y,y}+N_{\mathrm{xy},x}+{\left(N_{x}v_{,x}\right)}_{,x}=\;0\\ M_{x,\mathrm{xx}}+M_{y,\mathrm{yy}}+{2M}_{\mathrm{xy},\mathrm{xy}}+{\left(N_{x}w_{,x}\right)}_{,x}+{\left(N_{y}w_{,y}\right)}_{,y}+{\left(N_{\mathrm{xy}}w_{,x}\right)}_{,y}+{\left(N_{\mathrm{xy}}w_{,y}\right)}_{,x}=\;0 \end{array}$$

and are the basis for describing the behaviour of the plate structure. To solve the problem, boundary conditions should be defined.

For x = const., boundary conditions are:

(7) $$\begin{array}{l} \int_{0}^{b}{(N}_{x}-\;t{\cdot p}^{0})\;\delta \mathrm{udy}=0\to \int_{0}^{b}{(N}_{x}\;–{N_{x}}^{0})\;\delta \mathrm{udy}=0\\ \int_{0}^{b}N_{\mathrm{xy}}\delta v\mathrm{dy}=0\\ \int_{0}^{b}M_{x}\delta w_{,x}\mathrm{dy}=0\\ \int_{0}^{b}\left(M_{x,x}+2M_{\mathrm{xy},y}+N_{x}w_{,x}+N_{\mathrm{xy}}w_{,y}\right)\delta \mathrm{wdy}=0 \end{array}$$

where ${N_{x}}^{0}$ is the pre-buckling load at the edge of the single plate.

For y = const., boundary conditions are:

(8a) $$\begin{array}{l} \int_{0}^{a}N_{\mathrm{xy}}\delta \mathrm{udx}=0\\ \int_{0}^{a}{(N}_{y}+N_{y}v_{,y})\delta \mathrm{vdx}=0\\ \int_{0}^{a}M_{y}\delta w_{,y}\mathrm{dx}=0\\ \int_{0}^{a}\left(M_{y,y}+2M_{\mathrm{xy},x}+N_{y}w_{,y}+N_{\mathrm{xy}}w_{,x}\right)\delta \mathrm{wdx}=0 \end{array}$$

for a single plate. After the integration of the third equilibrium equation, we obtain the last boundary condition for x and y = const.:

(8b) $$2M_{\mathrm{xy}}\delta w=0$$

for each corner of a single plate.

**Figure 2:**
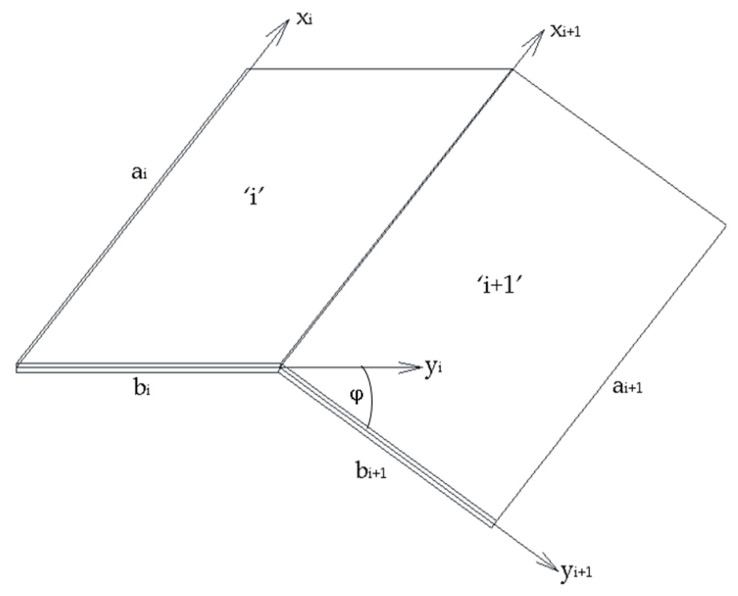
Two adjacent plates with local coordinate systems and main dimensions.

Then, the continuity conditions are formulated between two single plates (see Figure 2). The equations are presented only for the eccentricity about the major axis, and are as follows:

(9) $$\begin{array}{l} u_{i+1}\left|0\right.=u_{i}\left|+\right.\\ v_{i+1}\left|0\right.=w_{i}\left|+\right.\sin\left(\varphi \right)+\;v_{i}\left|+\right.\cos\left(\varphi \right)\\ w_{i+1}\left|0\right.=w_{i}\left|+\right.\cos\left(\varphi \right)-v_{i}\left|+\right.\sin\left(\varphi \right)\\ w_{i+1,y}\left|0\right.=w_{i,y}\left|+\right.\\ M_{(i+1)y}\left|0\right.=M_{\mathrm{iy}}\left|+\right.\\ N_{(i+1)y}\left|0\right.+N_{(i+1)y}\left|0v_{(i+1)y}\right.\left|0\right.-\left(N_{\mathrm{iy}}\left|+\right.+N_{\mathrm{iy}}\left|+\right.v_{i,y}\left|+\right.\right)\cos\left(\varphi \right)\\ -(M_{iy,y}\left|+\right.+2M_{\mathrm{ixy},x}\left|+\right.+N_{iy}\left|+\right.w_{i,y}\left|+\right.+N_{\mathrm{ixy}}\left|+\right.w_{i,x}\left|+\right.)\sin\left(\varphi \right)\\ \begin{array}{l} \left(M_{(i+1)y,y}\left|0\right.+2M_{(i+1)\mathrm{xy},x}\left|0\right.+N_{(i+1)y}\left|0\right.w_{(i+1),y}\left|0\right.+N_{(i+1)\mathrm{xy}}\left|0\right.w_{(i+1),x}\left|0\right.\right)\\ +\left(N_{\mathrm{iy}}\left|+\right.+N_{\mathrm{iy}}\left|+\right.v_{I,y}\left|+\right.\right)\sin\left(\varphi \right)\\ -\left(M_{\mathrm{iy},y}\left|+\right.+2M_{\mathrm{ixy},x}\left|+\right.+N_{\mathrm{iy}}\left|+\right.w_{i,y}\left|+\right.+N_{\mathrm{ixy}}\left|+\right.w_{i,x}\left|+\right.\right)\cos\left(\varphi \right)=0 \end{array}\\ N_{(i+1)\mathrm{xy}}\left|0\right.=N_{\mathrm{ixy}}\left|+\right. \end{array}$$

where $\left|0\right.\;\mathrm{means}\;y_{i+1}=0$, $\left|+\right.\;\mathrm{means}\;y_{i}=b_{i}$, and φ is the angle between two plates (see Figure 2).

#### 2.1.1. Boundary Conditions for Buckling Problem

The buckling problem can be solved if boundary conditions are written in the following form (for the eccentricity about the major axis).

- For x = 0, L:

(10) $$\begin{array}{l} u_{i}=f\left(y\right)\left|x=0\right.\;or\;g\left(y\right)\left|x=L\right.\\ v_{i}=0\\ M_{\mathrm{ix}}=0\\ w_{i}=0\\ \sum \int_{0}^{b_{i}}N_{\mathrm{ix}}\mathrm{dy}=\sum b_{i}{N_{\mathrm{ix}}}^{0}=F\;(\mathrm{for}\;x=L)\\ \sum \int_{0}^{b_{i}}N_{\mathrm{ix}}y_{i}\mathrm{dy}=F\cdot e\;(\mathrm{for}\;x=L) \end{array}$$

- For y = 0, $b_{i}$ (b.c. concern only lips):

(11) $$\begin{array}{l} N_{\mathrm{ixy}}=0\\ N_{\mathrm{iy}}=0\\ M_{\mathrm{iy}}=0\\ M_{\mathrm{iy},y}+2M_{\mathrm{ixy},x}=0 \end{array}$$

where i is the i-th wall of the member and L is the total length of the member.

Conditions of continuity (static conditions) are taken into consideration only in the linear form.

#### 2.1.2. Boundary Conditions for Post-Buckling Problem

In order to solve the problem of the post-buckling behaviour (nonlinear stability problem), boundary conditions had to be formulated again and are written below (for the eccentricity about the major axis):

- For x = 0, L:

(12) $$\begin{array}{l} \sum \int_{0}^{b_{i}}N_{\mathrm{ix}}\mathrm{dy}=\sum b_{i}{N_{\mathrm{ix}}}^{0}=F\;(\mathrm{for}\;x=L)\\ \sum \int_{0}^{b_{i}}N_{\mathrm{ix}}y_{i}\mathrm{dy}=F\cdot e\;(\mathrm{for}\;x=L)\\ v_{i}=0\\ M_{\mathrm{ix}}=0\\ w_{i}=0 \end{array}$$

- For y = 0, $b_{i}$ (boundary conditions concern only lips):

(13) $$\begin{array}{l} N_{\mathrm{ixy}}=0\\ {(N}_{\mathrm{iy}}+N_{\mathrm{iy}}v_{i,y})=0\\ M_{\mathrm{iy}}=0\\ M_{\mathrm{iy},y}+2M_{\mathrm{ixy},x}+N_{\mathrm{iy}}w_{i,y}+N_{\mathrm{ixy}}w_{i,x}=0 \end{array}$$

where e is the eccentricity, F is the eccentric compression force.

### 2.2. Finite Difference Method

**Figure 3:**
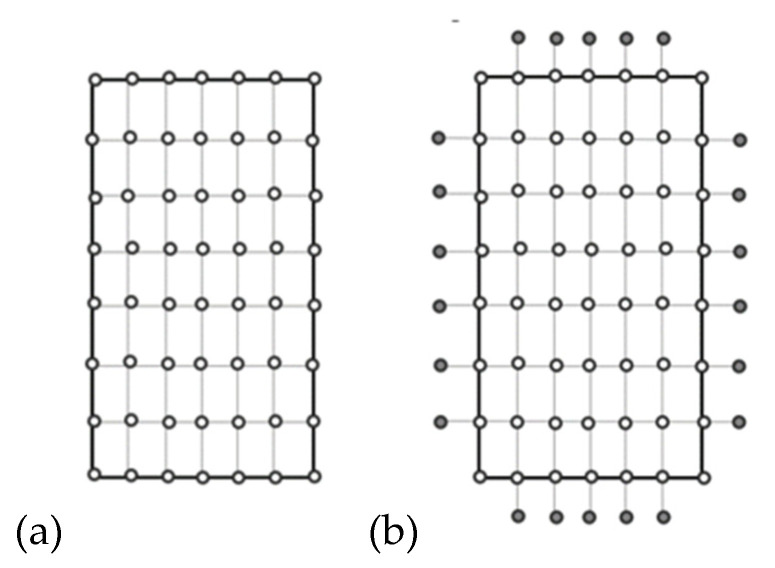
Mesh of nodes on single plate (wall): (**a**) for ‘u’ and ‘v’ displacements; (**b**) for ‘w’ displacement (○—active node, ●—passive node).

In order to solve equilibrium Equations (6a) or (6b), the finite difference method (FDM) was applied. First, each wall of the member was divided into nodes (the displacement ‘w’ is calculated in the active and passive nodes, but the displacements ‘u’ and ‘v’ are calculated only in the active nodes; see Figure 3).

The mesh shown in Figure 3 is adequate for the difference quotients that appear in the equilibrium equations.

**Figure 4:**
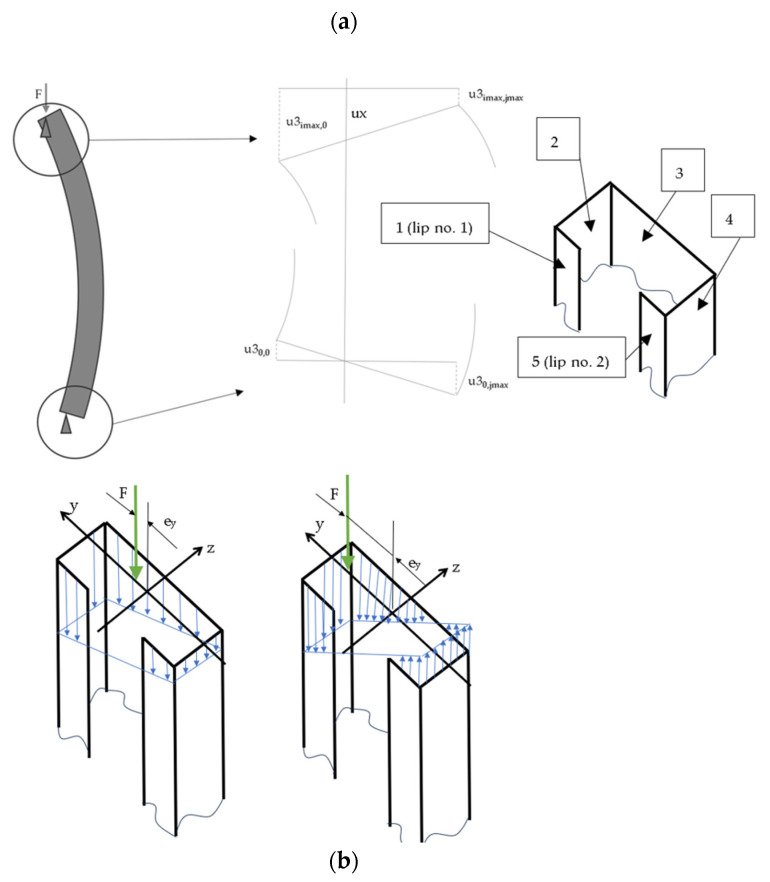
Isometric view of analysed member: (**a**) displacements (ux, ${u3}_{\mathrm{imax},jmax}$, ${u3}_{\mathrm{imax},0}$, ${u3}_{0,0}$, ${u3}_{0,\mathrm{jmax}}$) and numbering of walls (1 to 5); (**b**) stress distribution (blue arrows) for small and large eccentricities e_y_ (F—eccentric force, e_y_—eccentricity measured from ‘z’ axis).

**Figure 5:**
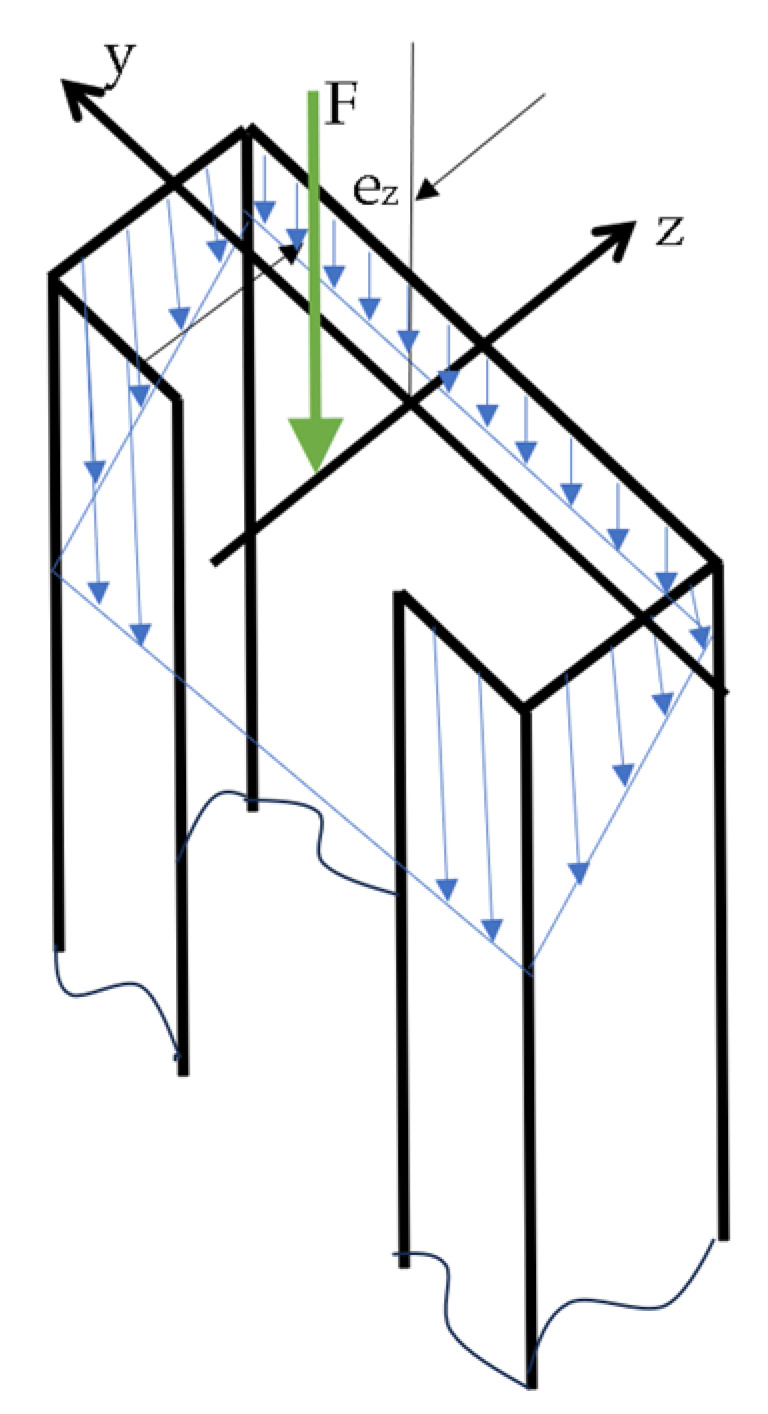
Exemplary stress distribution (blue arrows) for eccentricity measured from ‘y’ axis (e_z_ > 0). Central difference quotients were used for discretization of equilibrium equations but left- and right-side difference quotients were applied to boundary conditions and conditions of continuity. The difference quotient formulae are written in Appendix A.

Boundary conditions expressed by difference quotients are described below. They were written at two edges of lips, two edges of flanges, and two edges of the web of the analysed member, simply supported at the ends. Schemes of deformation of the structure and stress distribution are shown in Figure 4 and Figure 5.

#### 2.2.1. Solution of the Linear Buckling Problem

The boundary conditions for the buckling state of the member are written under the assumption that in the buckling state, displacements ${u3}_{\mathrm{imax},\mathrm{jmax}}$, ${u3}_{\mathrm{imax},0}$, ${u3}_{0,0}$, and ${u3}_{0,\mathrm{jmax}}$ are equal to zero, because the buckling problem is an eigenvalue problem.

Moreover, angles of rotation of the top and bottom cross section of the member are assumed to be the same.

In the case of linear stability (buckling state), linear equilibrium equations were formulated. The eigenvalue problem was solved using the following equations:

(14) $$\begin{array}{l} C\left(Nx0,u,v,w\right)=0\\ A=C\left(0,u,v,w\right)\\ B=A-C\\ D=A^{-1}B\\ Nx0=1/D1[[k]] \end{array}$$

where k means the k-th element of matrix **D1** (k = 1 usually corresponds to the buckling load), matrix **C** means simultaneous equilibrium equations with boundary conditions, matrix **A** is equal to matrix **C** taking into account Nx0 = 0, and matrix **D1** is the matrix of eigenvalues of matrix **D**.

For eccentricity about the major axis, boundary conditions are:

- For x = 0, L (or i = 0 or imax):

(15) $$\begin{array}{l} {u3}_{\mathrm{imax},0}=-(e+b3/2)/(-e11+b3/2)({u3}_{\mathrm{imax},\mathrm{jmax}})+\mathrm{ux}\cdot b3/(-e+b3/2)\\ {u1}_{\mathrm{imax},0}=(b1-j1\cdot \mathrm{hy}1)({u3}_{\mathrm{imax},\mathrm{jmax}}-{u3}_{\mathrm{imax},0})/b3+{u3}_{\mathrm{imax},0}\\ {u2}_{\mathrm{imax},j}={u3}_{\mathrm{imax},0}\\ {u3}_{\mathrm{imax},0}=-(e+b3/2)/(-e+b3/2)\cdot ({u3}_{\mathrm{imax},\mathrm{jmax}})+\mathrm{ux}\cdot b3/(-e+b3/2)\\ {u4}_{\mathrm{imax},j}={u3}_{\mathrm{imax},\mathrm{jmax}} \end{array}$$

- For y = 0, $b_{i}$ (it concerns only two lips—j = 0 or jmax):

(16) $$\begin{array}{l} {N1}_{(i,j)\mathrm{xy}}={N5}_{(i,j)\mathrm{xy}}=0\\ {N1}_{(i,j)y}={N5}_{(i,j)y}=0\\ {M1}_{(i,j)y}={M5}_{(i,j)y}=0\\ {M1}_{(i,j)y,y}+2{M1}_{(i,j)\mathrm{xy},x}={M5}_{(i,j)y,y}+2{M5}_{(i,j)\mathrm{xy},x}=0 \end{array}$$

where i, j are the coordinates of the node in the longitudinal and perpendicular directions, respectively, imax is the maximum number of nodes in the longitudinal direction, and jmax is the maximum number of nodes in the perpendicular direction.

Boundary conditions for eccentricity about the minor axis can be written in the following form:

- For x = 0:

(17) $$\begin{array}{c} {u11}_{0,j}={u11}_{0,\mathrm{jmax}}\\ {u12}_{0,j}=\left({u13}_{0,\mathrm{jmax}}-{u11}_{0,\mathrm{jmax}}\right)\cdot j/\mathrm{jmax}+{u11}_{0,\mathrm{jmax}}\\ {u13}_{0,j}={u13}_{0,\mathrm{jmax}}\\ {u14}_{0,j}=\left({u11}_{0,\mathrm{jmax}}-{u13}_{0,\mathrm{jmax}}\right)\cdot j/\mathrm{jmax}+{u13}_{0,\mathrm{jmax}}\\ {u15}_{0,j}={u11}_{0,\mathrm{jmax}}\\ {M1}_{(i,j)x}={M2}_{(i,j)x}={M3}_{(i,j)x}={M4}_{(i,j)x}={M5}_{(i,j)x}=0\\ \;{w11}_{i,j}={w12}_{i,j}={w13}_{i,j}={w14}_{i,j}={w15}_{i,j}=0 \end{array}$$

- For x = L:

(18) $$\begin{array}{l} {u11}_{\mathrm{imax},j}={u11}_{\mathrm{imax},\mathrm{jmax}},\\ {u12}_{\mathrm{imax},j}={u11}_{\mathrm{imax},\mathrm{jmax}}-j/\mathrm{jmax}\cdot \left({u11}_{\mathrm{imax},\mathrm{jmax}}-{u13}_{\mathrm{imax},\mathrm{jmax}}\right),\\ {u13}_{\mathrm{imax},j}={u13}_{\mathrm{imax},\mathrm{jmax}},\\ {u14}_{\mathrm{imax},j}={u13}_{\mathrm{imax},\mathrm{jmax}}-j/\mathrm{jmax}\cdot \left({u13}_{\mathrm{imax},\mathrm{jmax}}-{u11}_{\mathrm{imax},\mathrm{jmax}}\right)\\ {u15}_{\mathrm{imax},j}={u11}_{\mathrm{imax},\mathrm{jmax}}\\ \sum \int_{0}^{b_{i}}N_{\mathrm{ix}}\mathrm{dy}=F\;(\mathrm{for}\;x=L)\\ \sum \int_{0}^{b_{i}}N_{\mathrm{ix}}z_{i}(y_{i})\mathrm{dy}=F\cdot ez\;(\mathrm{for}\;x=L) \end{array}$$

- For y = 0, $b_{i}$ (it concerns only two lips—j = 0 or jmax):

(19) $$\begin{array}{l} {N1}_{(i,j)\mathrm{xy}}={N5}_{(i,j)\mathrm{xy}}=0\\ {N1}_{(i,j)y}={N5}_{(i,j)y}=0\\ {M1}_{(i,j)y}={M5}_{(i,j)y}=0\\ {M1}_{(i,j)y,y}+2{M1}_{(i,j)\mathrm{xy},x}={M5}_{(i,j)y,y}+2{M5}_{(i,j)\mathrm{xy},x}=0 \end{array}$$

For the Equations (15)–(19), the following explanations are added:

- u1, u2, u3, u4, u5 are the mean displacements in x directions for plates no. 1,2,3,4,5 corresponding to eccentricity about the major axis (see Figure 4);
- u11, u12, u13, u14, u15 are the mean displacements in x direction for plates no. 1,2,3,4,5 corresponding to eccentricity about the minor axis (see Figure 4);
- M1, M2, M3, M4, M5 are the bending moments at the edges of plates no. 1,2,3,4,5.

#### 2.2.2. Solution of the Nonlinear Post-Buckling Problem

In this subsection, boundary conditions for the post-buckling state are defined for eccentricity about the major axis as follows.

- For x = L (top of member—i = imax):

(20) $$\begin{array}{l} {w1}_{\mathrm{imax},j}={w2}_{\mathrm{imax},j}={w3}_{\mathrm{imax},j}={w4}_{\mathrm{imax},j}={w5}_{\mathrm{imax},j}=\;0\\ {v1}_{\mathrm{imax},j}={\;v2}_{\mathrm{imax},j}={\;v3}_{\mathrm{imax},j}={\;v4}_{\mathrm{imax},j}={\;v5}_{\mathrm{imax},j}=\;0\\ {u3}_{\mathrm{imax},0}=-(e+b3/2)/(-e11+b3/2)({u3}_{\mathrm{imax},\mathrm{jmax}})+\mathrm{ux}\cdot b3/(-e+b3/2)\\ {u1}_{\mathrm{imax},0}=(b1-j1\cdot \mathrm{hy}1)({u3}_{\mathrm{imax},\mathrm{jmax}}-{u3}_{\mathrm{imax},0})/b3+{u3}_{\mathrm{imax},0}\\ {u2}_{\mathrm{imax},j}={u3}_{\mathrm{imax},0}\\ {u3}_{\mathrm{imax},0}=-(e+b3/2)/(-e+b3/2)\cdot ({u3}_{\mathrm{imax},\mathrm{jmax}})+\mathrm{ux}\cdot b3/(-e+b3/2)\\ {u4}_{\mathrm{imax},j}={u3}_{\mathrm{imax},\mathrm{jmax}}\\ {u5}_{\mathrm{imax},j}=\;(b3-j\cdot \mathrm{hy}1)\cdot ({u3}_{\mathrm{imax},\mathrm{jmax}}-{u3}_{\mathrm{imax},0})/b3+{u3}_{\mathrm{imax},0}\\ {M1}_{(i\max,j)x}={M2}_{(i\max,j)x}={M3}_{(i\max,j)x}={M4}_{(i\max,j)x}={M5}_{(i\max,j)x}=0\\ \sum \int_{0}^{b_{i}}N_{\mathrm{ix}}\mathrm{dy}=F\;(\mathrm{for}\;x=L)\\ \sum \int_{0}^{b_{i}}N_{\mathrm{ix}}y_{i}\mathrm{dy}=F\cdot ey\;(\mathrm{for}\;x=L) \end{array}$$

where ${u3}_{\mathrm{imax},\mathrm{jmax}}$ and ux are unknowns to be determined using the last two equations additionally.

- For x = 0 (bottom of member—i = 0):

(21) $$\begin{array}{l} {w1}_{0,j}={w2}_{0,j}={w3}_{0,j}={w4}_{0,j}={w5}_{0,j}=\;0\\ {v1}_{0,j}={\;v2}_{0,j}={\;v3}_{0,j}={\;v4}_{0,j}={\;v5}_{0,j}=\;0\\ {u3}_{0,0}=(b3/2+e)/(b3/2-e)({u3}_{\mathrm{imax},\mathrm{jmax}}-\mathrm{ux})\\ {u1}_{0,j}=\;(e+b3/2-b1+j\cdot \mathrm{hy}1)/(e+b3/2)\cdot {u3}_{0,0}\\ {u2}_{0,j}={u3}_{0,0}\\ {u3}_{0,j}=\;(e+b3/2-j\cdot \mathrm{hy}3)/(e+b3/2)\cdot {u3}_{0,0}\\ {u4}_{0,j}={u3}_{0,0}\cdot (e-b3/2)/(e+b3/2)\\ {u5}_{0,j}=(e-b3/2+j\cdot \mathrm{hy}1)/(e+b3/2)\cdot {u3}_{0,0}\\ {M1}_{(0,j)x}={M2}_{(0,j)x}={M3}_{(0,j)x}={M4}_{(0,j)x}={M5}_{(0,j)x}=0 \end{array}$$

- For y = 0 (lip 1 (wall 1)—free edge):

(22) $$\begin{array}{l} {N1}_{(i,0)\mathrm{xy}}=0\\ {N1}_{(i,0)y}+{N1}_{(i,0)y}{v1}_{(i,0),y}=0\\ {M1}_{(i,0)y}=0\\ {M1}_{(i,0)y,y}+2{M1}_{(i,0)\mathrm{xy},x}+{N1}_{(i,0)y}{w1}_{(i,0),y}+{N1}_{(i,0)\mathrm{xy}}{w1}_{(i,0),x}=0 \end{array}$$

- For y = b5 (lip 2 (wall 5)—free edge):

(23) $$\begin{array}{l} {N5}_{(i,\mathrm{jmax})\mathrm{xy}}=0\\ {N5}_{(i,\mathrm{jmax})y}+{N5}_{(i,\mathrm{jmax})y}{v5}_{(i,\mathrm{jmax}),y}\;=0\\ {M5}_{(i,\mathrm{jmax})y}=0\\ {M5}_{(I,\mathrm{jmax})y,y}+2{M1}_{(I,\mathrm{jmax})\mathrm{xy},x}+{N5}_{(I,\mathrm{jmax})y}{w5}_{(I,\mathrm{jmax}),y}+{N5}_{(I,\mathrm{jmax})\mathrm{xy}}{w5}_{(I,\mathrm{jmax}),x}=0 \end{array}$$

where i = 0, 1, 2 …imax.

For eccentricity about the minor axis, boundary conditions can be written as:

- For x = 0:

(24) $$\begin{array}{l} {u11}_{0,j}={u11}_{0,\mathrm{jmax}}\\ {u12}_{0,j}=\left({u13}_{0,\mathrm{jmax}}-{u11}_{0,\mathrm{jmax}}\right)\cdot j/\mathrm{jmax}+{u11}_{0,\mathrm{jmax}}\\ {u13}_{0,j}={u13}_{0,\mathrm{jmax}}\\ {u14}_{0,j}=\left({u11}_{0,\mathrm{jmax}}-{u13}_{0,\mathrm{jmax}}\right)\cdot j/\mathrm{jmax}+{u13}_{0,\mathrm{jmax}}\\ {u15}_{0,j}={u11}_{0,\mathrm{jmax}}\\ {M1}_{(i,j)x}={M2}_{(i,j)x}={M3}_{(i,j)x}={M4}_{(i,j)x}={M5}_{(i,j)x}=0\\ {w11}_{i,j}={w12}_{i,j}={w13}_{i,j}={w14}_{i,j}={w15}_{i,j}=0 \end{array}$$

- For x = L:

(25) $$\begin{array}{l} {u11}_{\mathrm{imax},j}={u11}_{\mathrm{imax},\mathrm{jmax}}\\ {u12}_{\mathrm{imax},j}={u11}_{\mathrm{imax},\mathrm{jmax}}-j/\mathrm{jmax}\cdot \left({u11}_{\mathrm{imax},\mathrm{jmax}}-{u13}_{\mathrm{imax},\mathrm{jmax}}\right)\\ {u13}_{\mathrm{imax},j}={u13}_{\mathrm{imax},\mathrm{jmax}}\\ {u14}_{\mathrm{imax},j}={u13}_{\mathrm{imax},\mathrm{jmax}}-j/\mathrm{jmax}\cdot \left({u13}_{\mathrm{imax},\mathrm{jmax}}-{u11}_{\mathrm{imax},\mathrm{jmax}}\right)\\ {u15}_{\mathrm{imax},j}={u11}_{\mathrm{imax},\mathrm{jmax}}\\ \sum \int_{0}^{b_{i}}N_{\mathrm{ix}}\mathrm{dy}=F\;(\mathrm{for}\;x=L)\\ \sum \int_{0}^{b_{i}}N_{\mathrm{ix}}z_{i}(y_{i})\mathrm{dy}=F\cdot ez\;(\mathrm{for}\;x=L) \end{array}$$

- For y = 0 (lip 1 (wall 1)—free edge):

(26) $$\begin{array}{l} {N1}_{(i,0)\mathrm{xy}}=0\\ {N1}_{(i,0)y}+{N1}_{(i,0)y}{v1}_{(i,0),y}=0\\ {M1}_{(i,0)y}=0\\ {M1}_{(I,0)y,y}+2{M1}_{(I,0)\mathrm{xy},x}+{N1}_{(I,0)y}{w1}_{(I,0),y}+\\ {N1}_{(i,0)\mathrm{xy}}{w1}_{(i,0),x}=0 \end{array}$$

- For y = b5 (lip 2 (wall 5)—free edge):

(27) $$\begin{array}{l} {N5}_{(i,\mathrm{jmax})\mathrm{xy}}=0\\ {N5}_{(i,\mathrm{jmax})y}+{N5}_{(i,\mathrm{jmax})y}{v5}_{(i,\mathrm{jmax}),y}\;=0\\ {M5}_{(i,\mathrm{jmax})y}=0\\ {M5}_{(i,\mathrm{jmax})y,y}+2{M1}_{(i,\mathrm{jmax})\mathrm{xy},x}+{N5}_{(i,\mathrm{jmax})y}{w5}_{(i,\mathrm{jmax}),y}+{N5}_{(i,\mathrm{jmax})\mathrm{xy}}{w5}_{(i,\mathrm{jmax}),x}=0 \end{array}$$

Moreover, angles of rotation of the top and bottom cross sections of the member are assumed to be the same.

#### 2.2.3. Equilibrium Equations and Definition of Internal, Sectional Pre-Buckling Forces—Major Axis

The equilibrium equations for pre- and post-buckling states are shown for a single plate in Appendix B in discrete form. In the third equation, an internal, sectional pre-buckling load appears. The internal pre-buckling forces are presented in Appendix D.

### 2.3. Solution of Nonlinear Algebraic Equations

In order to solve the equations derived in Chapter 3, the Newton–Raphson iterative method was implemented using the Object Pascal programming language. The algorithm works in the load substep loop and the external load step loop (with a maximum number of load steps), and can be written below in general form [19]:

(28) $$x_{i+1,j}=x_{i,j}-J_{i,j}^{-1}(x)\cdot F_{i,j}(x)$$

where **x** is the vector of unknowns, **J** is the Jacoby’s matrix, **F** is the vector of the left side of algebraic equations (**F** = **0**), i is the i-th iteration (i = 0, 1, 2…), and j is the j-th load step. For i = 0, initial vector **x**_0_ =0.

The criterion to stop the Newton–Raphson algorithm (28) (for the internal loop) was proposed as:

(29) $$\sum_{i=1}^{N}{|F}_{i,j}|\;<\;\frac{N}{\mathrm{maximum}\;\mathrm{number}\;\mathrm{of}\;\mathrm{load}\;\mathrm{steps}}$$

where F_i_ is the left side of the i-th algebraic equation (F_i_ = 0), N is the number of algebraic equations, and i = 1,2, …, N.

**Figure A1:**
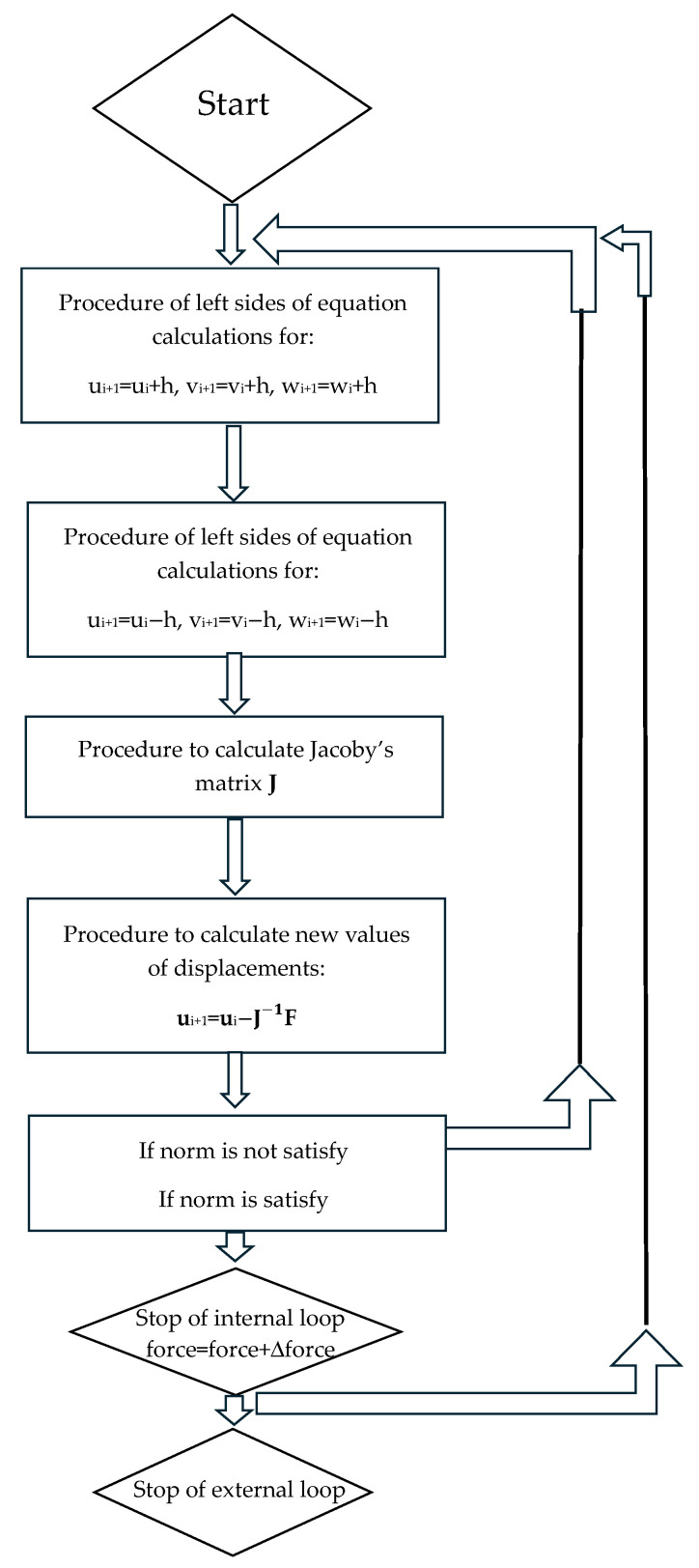
Algorithm of working of authorial computer program, where jmax is the maximal value of load steps and u, v, and w are the displacements.

The algorithm is shown in Appendix C in Figure A1. If the calculation procedure finds the point in the equilibrium path at which the Newton–Raphson procedure does not work correctly, a special algorithm is switched on to obtain the correct displacement value. This algorithm works using the checking procedure norm. If the norm satisfies inequality (29), then the vector of unknowns is correct, but if the unknowns are not satisfied, then the previous vector (from the previous iteration) is added to this scaled vector. The scaling factor is calculated as:

(30) $$\mathrm{Factor}=F_{\max}/((\sum_{i=1}^{N}{|F}_{i,j}|)(j3+1))$$

where j3 is the number of the internal loops of the algorithm and F_max_ is the maximum value of the left side of the system of algebraic equations. In order to overcome obstacles like bifurcation points (change of mode of buckling), an additional procedure was introduced. The procedure is described in mathematical form:

(31) $$x_{i+1,j}=x_{i}+2\cdot \left(\mathrm{Random}-\frac{1}{2}\right)\cdot \mathrm{factor}\cdot t$$

where the ‘Random’ function is a function that is equal to 0 to 1 and is a number of pseudo-random type, while t is the thickness. If the factor is greater than 0.001, then it always equals 0.001. In some cases, in the algorithm uses an iteration step: when the force increases with a constant value (force control), the step goes down and the algorithm above (30) is used.

## 3. Experimental Study

The eccentric compression test in the case of buckling analysis is a basis of information to validate numerical models. In this validation, the main results were compared with the results of the tests [7,8]. A special stand (grip) was installed on the test machine (Instron—maximum load 200 kN). Furthermore, despite the measurement system integrated with the machine, the ARAMIS digital image correlation system (DIC) was adopted to measure the field of displacements and strains. The loading velocity was 1 mm/min, so the test was carried out as quasi-static. Tests were carried out for a wide range of eccentricities.

**Figure 6:**
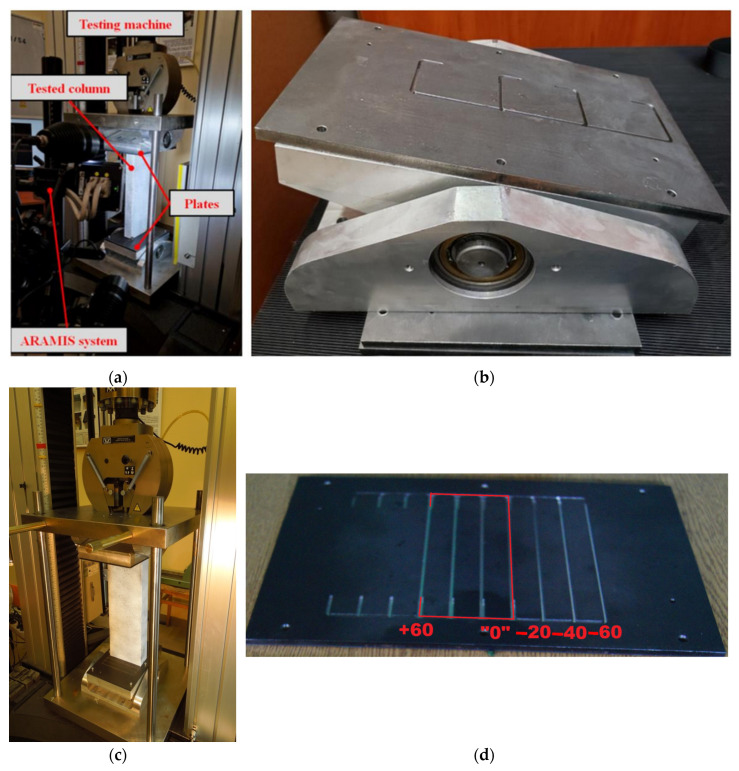
Views of experimental stands: (**a**) general view of the stand to conduct eccentric compression tests (eccentricity about major axis), (**b**) plate with grooves to establish eccentricity [8], (**c**) general view of the stand to conduct eccentric compression test (eccentricity about minor axis) [7], (**d**) plate with grooves to establish eccentricity [7].

In Figure 6, stands to conduct experimental tests for eccentricity about minor and major axes are presented. The specimen was placed between two rigid plates. The bottom plate can only rotate (it has one degree of freedom), but the top plate can rotate and move in the load direction (two degrees of freedom are ensured). The bottom plates were produced with grooves that induce a certain eccentricity with respect to a certain axis.

**Figure 7:**
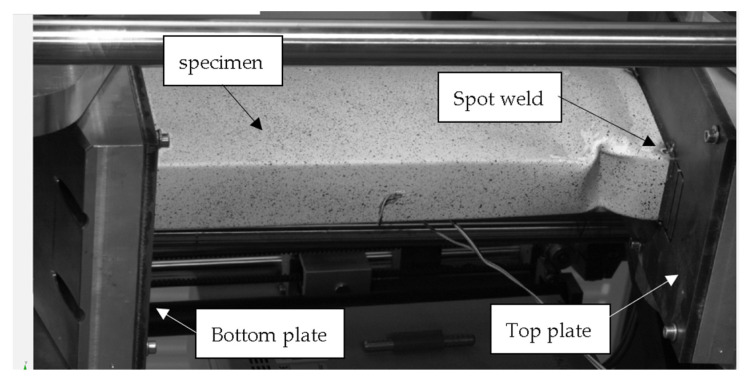
Exemplary view of the specimen from the DIC system—eccentricity about the major axis.

In Figure 7, the stand for performing the eccentric compression test is presented together with a description of the elements belonging to the stand.

**Figure 8:**
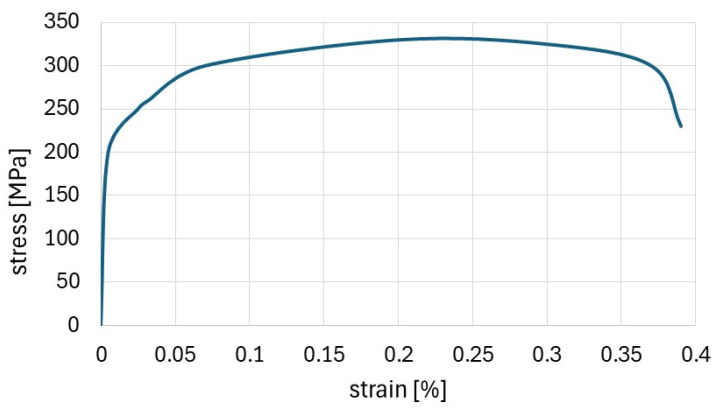
Diagram from tensile test [7].

The properties were determined from tensile tests performed on the coupons cut from the raw material. Detailed data on the material are given in [7,20] for members subjected to eccentric compression about minor and major axes, respectively. An exemplar stress–strain diagram of the tensile test for the member material (member subject to eccentric compression about the minor axis) is presented in Figure 8.

## 4. Numerical and Experimental Results

**Figure A2:**
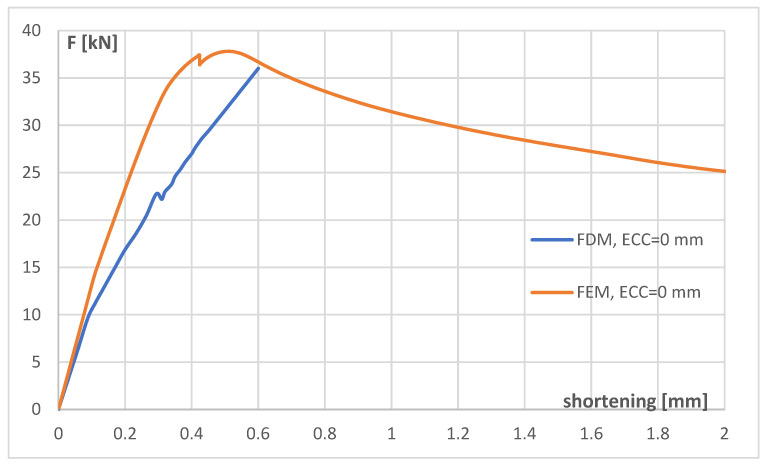
Validation of FD model for eccentricity = 0 (only axial compression).

A validation of the numerical FD model was performed and is shown in Appendix E (Figure A2) for the simple case of the member loading (eccentricity equals 0).

The buckling forces, for the eccentricity (about the major axis) of 15–60 mm, are shown in Table 1. The table shows selected experimental values obtained for the eccentricity about the major axis (F_ey,E_) and numerical values obtained from the FDM from linear (F_ey,L_) and nonlinear (F_ey,NL_) stability analysis.

**Figure 9:**
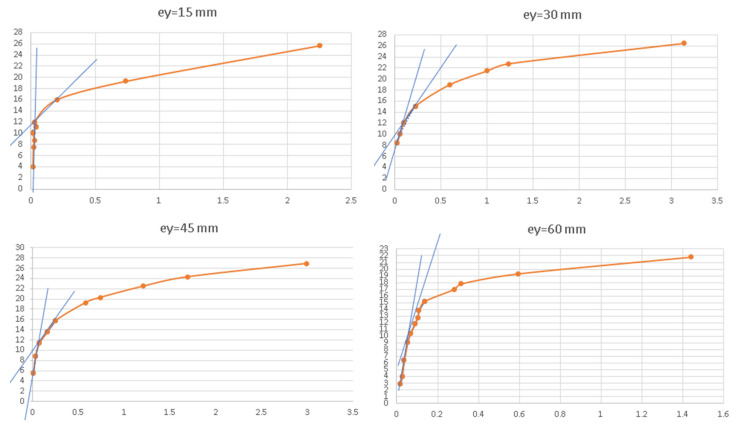
Modified method of determination buckling forces based on curves F vs. w^2^ (w—displacement) from experimental data (e_y_—eccentricity).

The experimental values of the buckling forces were obtained using a modified F-w^2^ method, which is explained in Figure 9. Displacement (deflection w) was recorded using the ARAMIS system (see the orange lines). It was taken from the point of the web at which the maximum deflection appeared. The point of cross section of the two secant lines designates the buckling force (see the blue lines). The buckling forces F_ey,L_ are eigenvalues obtained from the linear FDM analysis. The buckling forces on the basis of the nonlinear analysis of the FDM were obtained as an ordinate of the transition point from the pre-buckling to the post-buckling path. This method should start from drawings of the points (F, w2), where wis the amplitude of deflection. Next, for three points, the Pearson coefficient is calculated. After that, for four points, the coefficient is calculated again, but the mean value r_mean from two Pearson coefficients ‘r’ and the standard deviation Sn are calculated. If two values of ‘r’ are in the range of r_mean ± Sn·ta,k, we choose a group of points with the maximum ‘r’. This process is repeated up to the last point.

**Figure 10:**
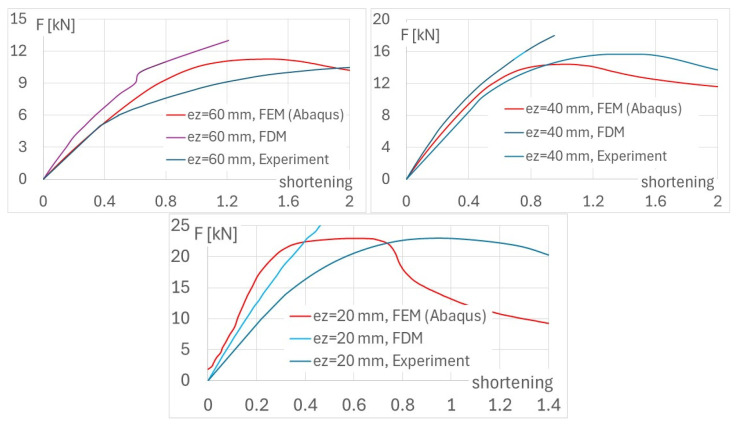
Diagrams of force vs. shortening from numerical methods and experiment for various eccentricity; FDM—finite difference method, FEM—finite element method, e_z_—eccentricity measured relative to minor axis.

The pre- and post-buckling equilibrium paths, obtained numerically (using the FDM, without post-ultimate phenomenon) and compared to the FEM and experimental data (Figure 10), are presented below for eccentricity about the minor axis.

**Figure 11:**
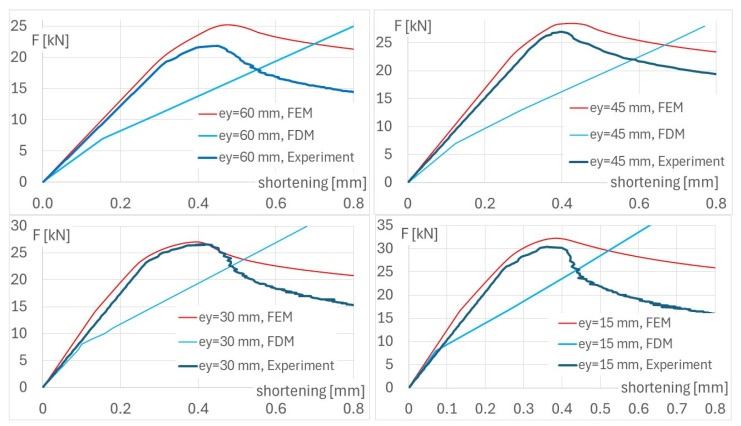
Diagrams of force vs. shortening from experiment and numerical methods; FDM—finite difference method, FEM—finite element method, e_y_—eccentricity about major axis.

Force-shortening diagrams for eccentricity about the major axis are shown in Figure 11. The paths of equilibrium were obtained from the FDM and compared with the experimental data and the FEM [20].

**Figure 12:**
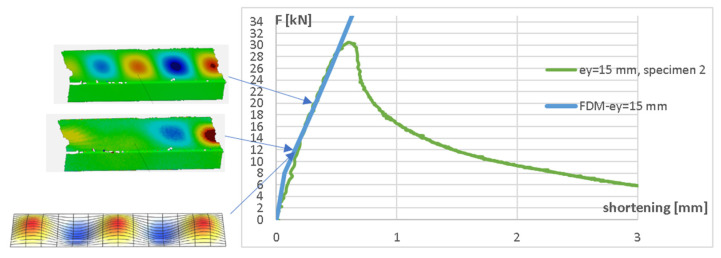
Buckling modes for compressive force F = 11 kN (FDM and DIC) and F = 17 kN (DIC) for eccentricity e_y_ = 60 mm.

**Figure 13:**
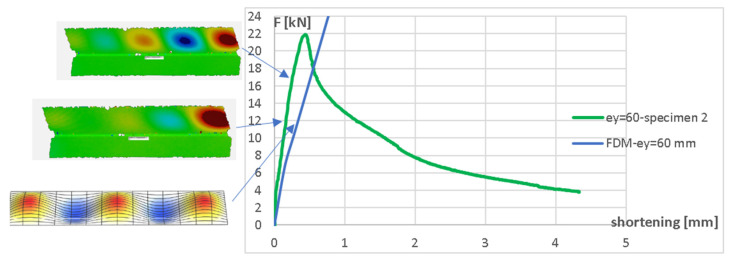
Buckling modes for compressive forces F = 12 kN (FDM and DIC) and 20 kN (DIC) for eccentricity e_y_ = 15 mm.

In order to validate the FDM algorithm more deeply, the analysis of buckling modes on the basis of both numerical (FDM) results and the experiment (deformation fields obtained from DIC Aramis system) was performed. Figure 12 shows the comparison of the buckling modes and equilibrium paths obtained from the experiment and the FDM for the eccentricity e_y_ = 60 mm, while Figure 13 shows the buckling modes and equilibrium paths for the eccentricity e_y_ = 15 mm (both eccentricities about the major axis).

The discrepancies between the experimental and numerical results, which are shown in Figure 10 and Figure 11, as well as in Figure A2 (between FDM and FE results), are due to several factors, i.e., imperfection distributions and small differences in boundary conditions that are assumed in numerical models, especially in FD models (about minor and major axes). In the FEM analysis, the master node was applied. The boundary conditions in displacements and rotations are distributed from master node to slave nodes at the ends of the member, while in the FDM, boundary conditions are assumed in displacements and forces as functions of unknown force of eccentric compression and maximum values of unknown displacements in edges. They can differ from the real boundary conditions, and they differ in the FDM and FEM models. This problem of the influence of boundary conditions in numerical models on the results will be investigated in the next paper.

The arrows indicate the force level corresponding to the particular buckling mode.

## 5. Conclusions

This paper presents a semi-analytical solution of the buckling and post-buckling problem concerning TWCFS lipped channel section members subject to eccentric compression about both the minor and major principal axes. The solution was based on the theory of thin plates. The equations of equilibrium of the section walls (thin plates) were derived from the principle of stationary total potential energy. Then, in order to solve the problem, the finite difference method (FDM) and Newton–Raphson method were adopted. FE code has to include a solution method based on nonlinear algebraic equations, i.e., the Newton–Raphson method. Application of the FDM allowed the modification of the calculation process using the Newton–Raphson method, and this modification was conducted.

Linear (buckling) and nonlinear (post-buckling) analysis was performed. As a result, the equilibrium paths of the pre- and post-buckling were determined. Comparison of the obtained numerical results based on the FDM, FEM simulation results, and experimental test results was carried out.

Good agreement of FDM and FEM results, as well as results of experiments, was obtained for members subjected to eccentric compression about the minor axis. Some discrepancies are observed in the case of major axis bending. For both types of eccentricities, good agreement was achieved in buckling modes, pre-buckling paths of equilibrium and buckling forces, especially between experiment data and numerical FDM analysis (linear). Worse agreement (in some cases) was achieved between FEM and experimental post-buckling paths and those obtained using the presented FDM method. Those discrepancies come mainly from differences in assumed boundary conditions.

Generally, the presented original analytical–numerical approach based on the FDM is competitive in comparison with FEM simulations. The calculations are much less time-consuming. It may also provide an upper-bound estimate of load-carrying capacity as an intersection of the post-buckling path obtained using the present method and the post-ultimate path obtained based on the yield line analysis [21]. This approach, as mentioned in the Introduction, can be applied as a useful tool to improve standard predictions, e.g., to modify imperfection factors for interactive plastic–elastic buckling [22]. This will be an area of further research.

This aim cannot be achieved using FEM analysis, since FEM results cannot be generalised.

The research will also be extended on back-to-back members based on lipped channel section members with respect to material such as steel and composite and pulse loading (dynamic stability).

## Figures and Tables

**Table 1:** Buckling forces (eccentricity about major axis).

Eccentricity [mm]	F_ey,L_ [kN]	F_ey,NL_ [kN]	F_ey,E_ [kN]
15	13.1	8.1	12.5
30	13.0	8.0	11.9
45	12.8	7.5	11.5
60	12.5	7.0	9.0

## Data Availability

The original contributions presented in the study are included in the article, further inquiries can be directed to the corresponding author.

## Appendix A

In order to better explain how algebraic equations used in the algorithm for the solution of the buckling problem of the TWCS member are created, central left-sided and right-sided difference quotient definitions are shown.

- Central difference quotients:

(A1) $$\begin{array}{l} {u1}^{\left(1,0,0\right)}=\frac{{u1}_{i+1,j}-{u1}_{i-1,j}}{2\mathrm{hx}1},{v1}^{\left(1,0,0\right)}=\frac{{v1}_{i+1,j}-{v1}_{i-1,j}}{2\mathrm{hx}1},{w1}^{\left(1,0,0\right)}=\frac{{w1}_{i+1,j}-{w1}_{i-1,j}}{2\mathrm{hx}1},{u1}^{\left(2,0,0\right)}=\frac{{u1}_{i+1,j}-2{u1}_{i,j}+{u1}_{i-1,j}}{\mathrm{hx}1^{2}},\\ {v1}^{\left(2,0,0\right)}=\frac{{v1}_{i+1,j}-2{v1}_{i,j}+{v1}_{i-1,j}}{\mathrm{hx}1^{2}},{w1}^{\left(2,0,0\right)}=\frac{{w1}_{i+1,j}-2{w1}_{i,j}+{w1}_{i-1,j}}{\mathrm{hx}1^{2}},{u1}^{\left(0,1,0\right)}=\frac{{u1}_{i,j+1}-{u1}_{i,j-1}}{2\mathrm{hy}1},{v1}^{\left(0,1,0\right)}=\frac{{v1}_{i,j+1}-{v1}_{i,j-1}}{2\mathrm{hy}1},\\ {w1}^{\left(0,1,0\right)}=\frac{{w1}_{i,j+1}-{w1}_{i,j-1}}{2\mathrm{hy}1},{u1}^{\left(0,2,0\right)}=\frac{{u1}_{i,j+1}-2{u1}_{i,j}+{u1}_{i,j-1}}{\mathrm{hy}1^{2}},{v1}^{\left(0,2,0\right)}=\frac{{v1}_{i,j+1}-2{v1}_{i,j}+{v1}_{i,j-1}}{\mathrm{hy}1^{2}},\\ {w1}^{\left(0,2,0\right)}=\frac{{w1}_{i,j+1}-2{w1}_{i,j}+{w1}_{i,j-1}}{\mathrm{hy}1^{2}}\\ {u1}^{\left(1,1,0\right)}=\frac{{u1}_{i+1,j+1}-{u1}_{i+1,j-1}-{u1}_{i-1,j+1}+{u1}_{i-1,j-1}}{4\mathrm{hx}1\mathrm{hy}1},{v1}^{\left(1,1,0\right)}=\frac{{v1}_{i+1,j+1}-{v1}_{i+1,j-1}-{v1}_{i-1,j+1}+{v1}_{i-1,j-1}}{4\mathrm{hx}1\mathrm{hy}1},\\ {w1}^{\left(1,1,0\right)}=\frac{{w1}_{i+1,j+1}-{w1}_{i-1,j+1}+{w1}_{i-1,j-1}}{4\mathrm{hx}1\mathrm{hy}1},{w1}^{\left(2,1,0\right)}=\frac{{w1}_{i+1,j}-2{w1}_{i,j}+{w1}_{i-1,j}}{\mathrm{hx}1^{2}\mathrm{hy}1}-\frac{{w1}_{i+1,j-1}-2{w1}_{i,j-1}+{w1}_{i-1,j-1}}{\mathrm{hx}1^{2}\mathrm{hy}1},\\ {w1}^{\left(0,3,0\right)}=\frac{{w1}_{i,j+1}-2{w1}_{i,j}+{w1}_{i,j-1}}{\mathrm{hy}1^{3}}-\frac{{w1}_{i,j}-2{w1}_{i,j-1}+{w1}_{i,j-2}}{\mathrm{hy}1^{3}},{w1}^{\left(0,4,0\right)}=\frac{{w1}_{i,j+2}-4{w1}_{i,j+1}+6{w1}_{i,j}-4{w1}_{i,j-1}+{w1}_{i,j-2}}{\mathrm{hy}1^{4}},\\ {w1}^{\left(4,0,0\right)}=\frac{{w1}_{i+2,j}-4{w1}_{i+1,j}+6{w1}_{i,j}-4{w1}_{i-1,j}+{w1}_{i-2,j}}{\mathrm{hx}1^{4}},\\ {w1}^{\left(2,2,0\right)}=\frac{1}{\mathrm{hx}1^{2}\mathrm{hy}1^{2}}(4{w1}_{i,j}-2({w1}_{i,j+1}+{w1}_{i+1,j}+{w1}_{i,j-1}+{w1}_{i-1,j})+{w1}_{i+1,j+1}+{w1}_{i+1,j-1}+{w1}_{i-1,j-1}+{w1}_{i-1,j+1}) \end{array}$$

where hx1 and hy1 are the distances between the two nearest nodes in the ‘x’ and ‘y’ directions, and u1, v1, and w1 are the displacements of the middle surface of the wall in the ‘x’, ‘y’, and ‘z’ directions, respectively.

- Left-sided difference quotients:

(A2)
$$\begin{array}{l} {u1}^{\left(1,0,0\right)}=\frac{{u1}_{i+1,j}-{u1}_{i-1,j}}{2\mathrm{hx}1},{v1}^{\left(1,0,0\right)}=\frac{{v1}_{i+1,j}-{v1}_{i-1,j}}{2\mathrm{hx}1},{w1}^{\left(1,0,0\right)}=\frac{{w1}_{i+1,j}-{w1}_{i-1,j}}{2\mathrm{hx}1},{u1}^{\left(2,0,0\right)}=\frac{{u1}_{i+1,j}-2{u1}_{i,j}+{u1}_{i-1,j}}{\mathrm{hx}1^{2}},\\ {v1}^{\left(2,0,0\right)}=\frac{{v1}_{i+1,j}-2{v1}_{i,j}+{v1}_{i-1,j}}{\mathrm{hx}1^{2}},{w1}^{\left(2,0,0\right)}=\frac{{w1}_{i+1,j}-2{w1}_{i,j}+{w1}_{i-1,j}}{\mathrm{hx}1^{2}},{u1}^{\left(0,1,0\right)}=\frac{{u1}_{i,j}-{u1}_{i,j-1}}{\mathrm{hy}1},{v1}^{\left(0,1,0\right)}=\frac{{v1}_{i,j}-{v1}_{i,j-1}}{\mathrm{hy}1},\\ {w1}^{\left(0,1,0\right)}=\frac{{w1}_{i,j+1}-{w1}_{i,j-1}}{2\mathrm{hy}1},{u1}^{\left(0,2,0\right)}=\frac{{u1}_{i,j}-2{u1}_{i,j-1}+{u1}_{i,j-2}}{\mathrm{hy}1^{2}},{v1}^{\left(0,2,0\right)}=\frac{{v1}_{i,j}-2{v1}_{i,j-1}+{v1}_{i,j-2}}{\mathrm{hy}1^{2}},\\ {w1}^{\left(0,2,0\right)}=\frac{{w1}_{i,j+1}-2{w1}_{i,j}+{w1}_{i,j-1}}{\mathrm{hy}1^{2}},\\ {u1}^{\left(1,1,0\right)}=\frac{{u1}_{i+1,j}-{u1}_{i+1,j-1}-{u1}_{i-1,j}+{u1}_{i-1,j-1}}{2\mathrm{hx}1\mathrm{hy}1},\\ {v1}^{\left(1,1,0\right)}=\frac{{v1}_{i+1,j}-{v1}_{i+1,j-1}-{v1}_{i-1,j}+{v1}_{i-1,j-1}}{2\mathrm{hx}1\mathrm{hy}1},{w1}^{\left(1,1,0\right)}=\frac{{w1}_{i+1,j+1}-{w1}_{i+1,j-1}-{w1}_{i-1,j+1}+{w1}_{i-1,j-1}}{4\mathrm{hx}1\mathrm{hy}1},\\ {w1}^{\left(2,1,0\right)}=\frac{{w1}_{i+1,j}-2{w1}_{i,j}+{w1}_{i-1,j}}{\mathrm{hx}1^{2}\mathrm{hy}1}-\frac{{w1}_{i+1,j-1}-2{w1}_{i,j-1}+{w1}_{i-1,j-1}}{\mathrm{hx}1^{2}\mathrm{hy}1},{w1}^{\left(0,3,0\right)}=\frac{{w1}_{i,j+1}-2{w1}_{i,j}+{w1}_{i,j-1}}{\mathrm{hy}1^{3}}-\frac{{w1}_{i,j}-2{w1}_{i,j-1}+{w1}_{i,j-2}}{\mathrm{hy}1^{3}},\\ {w1}^{\left(0,4,0\right)}=\frac{{w1}_{i,j+2}-4{w1}_{i,j+1}+6{w1}_{i,j}-4{w1}_{i,j-1}+{w1}_{i,j-2}}{\mathrm{hy}1^{4}},{w1}^{\left(4,0,0\right)}=\frac{{w1}_{i+2,j}-4{w1}_{i+1,j}+6{w1}_{i,j}-4{w1}_{i-1,j}+{w1}_{i-2,j}}{\mathrm{hx}1^{4}},\\ {w1}^{\left(2,2,0\right)}=\frac{1}{\mathrm{hx}1^{2}\mathrm{hy}1^{2}}(4{w1}_{i,j}-2({w1}_{i,j+1}+{w1}_{i+1,j}+{w1}_{i,j-1}+{w1}_{i-1,j})+{w1}_{i+1,j+1}+{w1}_{i+1,j-1}+{w1}_{i-1,j-1}+{w1}_{i-1,j+1}) \end{array}$$

- Right-sided difference quotients:

(A3)
$$\begin{array}{l} {u2}^{\left(1,0,0\right)}=\frac{{u2}_{i+1,j1}-{u2}_{i-1,j1}}{2\mathrm{hx}1},{v2}^{\left(1,0,0\right)}=\frac{{v2}_{i+1,j1}-{v2}_{i-1,j1}}{2\mathrm{hx}1},{w2}^{\left(1,0,0\right)}=\frac{{w2}_{i+1,j1}-{w2}_{i-1,j1}}{2\mathrm{hx}1},{u2}^{\left(2,0,0\right)}=\frac{{u2}_{i+1,j1}-2{u2}_{i,j1}+{u2}_{i-1,j1}}{\mathrm{hx}1^{2}},\\ {v2}^{\left(2,0,0\right)}=\frac{{v2}_{i+1,j1}-2{v2}_{i,j1}+{v2}_{i-1,j1}}{\mathrm{hx}1^{2}},{w2}^{\left(2,0,0\right)}=\frac{{w2}_{i+1,j1}-2{w2}_{i,j1}+{w2}_{i-1,j1}}{\mathrm{hx}1^{2}},{u2}^{\left(0,1,0\right)}=\frac{{u2}_{i,j1+1}-{u2}_{i,j1}}{\mathrm{hy}2},{v2}^{\left(0,1,0\right)}=\frac{{v2}_{i,j1+1}-{v2}_{i,j1}}{\mathrm{hy}2},\\ {w2}^{\left(0,1,0\right)}=\frac{{w2}_{i,j1+1}-{w2}_{i,j1-1}}{2\mathrm{hy}2},{u2}^{\left(0,2,0\right)}=\frac{{u2}_{i,j1+2}-2{u2}_{i,j1+1}+{u2}_{i,j1}}{\mathrm{hy}2^{2}},{v2}^{\left(0,2,0\right)}=\frac{{v2}_{i,j1+1}-2{v2}_{i,j1+1}+{v2}_{i,j-1}}{\mathrm{hy}2^{2}},\\ {w2}^{\left(0,2,0\right)}=\frac{{w2}_{i,j1+1}-2{w2}_{i,j1}+{w2}_{i,j1-1}}{\mathrm{hy}2^{2}},\\ {u2}^{\left(1,1,0\right)}=\frac{{u2}_{i+1,j1+1}-{u2}_{i+1,j1}-{u2}_{i-1,j1+1}+{u2}_{i-1,j1}}{2\mathrm{hx}1\mathrm{hy}2},{v2}^{\left(1,1,0\right)}=\frac{{v2}_{i+1,j1+1}-{v2}_{i+1,j1}-{v2}_{i-1,j1+1}+{v2}_{i-1,j1}}{2\mathrm{hx}1\mathrm{hy}2},\\ {w2}^{\left(1,1,0\right)}=\frac{{w2}_{i+1,j1+1}-{w2}_{i+1,j1-1}-{w2}_{i-1,j1+1}+{w2}_{i-1,j1-1}}{4\mathrm{hx}1\mathrm{hy}2},{w2}^{\left(2,1,0\right)}=\frac{{w2}_{i+1,j1}-2{w2}_{i,j1}+{w2}_{i-1,j1}}{\mathrm{hx}1^{2\mathrm{hy}2}}-\frac{{w2}_{i+1,j1-1}-2{w2}_{i,j1-1}+{w2}_{i-1,j1-1}}{\mathrm{hx}1^{2\mathrm{hy}2}},\\ {w2}^{\left(0,3,0\right)}=\frac{{w2}_{i,j1+1}-2{w2}_{i,j1}+{w2}_{i,j1-1}}{\mathrm{hy}2^{3}}-\frac{{w2}_{i,j1}-2{w2}_{i,j1-1}+{w2}_{i,j1-2}}{\mathrm{hy}2^{3}},{w2}^{\left(0,4,0\right)}=\frac{{w2}_{i,j1+2}-4{w2}_{i,j1+1}+6{w2}_{i,j1}-4{w2}_{i,j1-1}+{w2}_{i,j1-2}}{\mathrm{hy}2^{4}},\\ {w2}^{\left(4,0,0\right)}=\frac{{w2}_{i+2,j1}-4{w2}_{i+1,j1}+6{w2}_{i,j1}-4{w2}_{i-1,j1}+{w2}_{i-2,j1}}{\mathrm{hx}1^{4}},{w2}^{\left(2,2,0\right)}=\frac{1}{\mathrm{hx}1^{2}\mathrm{hy}1^{2}}(4{w2}_{i,j1}-2({w2}_{i,j1+1}+{w2}_{i+1,j1}+{w2}_{i,j1-1}+{w2}_{i-1,j1})+{w2}_{i+1,j1+1}+{w2}_{i+1,j1-1}+{w2}_{i-1,j1-1}+{w2}_{i-1,j1+1}) \end{array}$$

## Appendix B

Three equilibrium equations for the lip (wall 1) are written in discrete form, as follows.

- First equation:

(K133∙(u11[i,−1 + j] − 2∙u11[i,j] + u11[i,1 + j]))/Power(hy1,2) +	(A4)
(K133∙(v11[−1 + i,−1 + j] − v11[−1 + i,1 + j] − v11[1 + i,−1 + j] + v11[1 + i,1 + j]))/(4∙hx1∙hy1) +	
(K133∙(w11[i,−1 + j] − 2∙w11[i,j] + w11[i,1 + j])∙(−w11[−1 + i,j] +	
+ w11[1 + i,j]))/(2∙hx1∙Power(hy1,2)) + (K133∙(−w11[i,−1 + j] + w11[i,1 + j])∙(w11[−1 + i,−1 + j] − w11[−1 + i,1 + j] − w11[1 + i,−1 + j] + w11[1 + i,1 + j]))/(8∙hx1∙Power(hy1,2)) + ((2∙K111∙(u11[−1 + i,j] − 2∙u11[i,j] + u11[1 + i,j]))/Power(hx1,2) + (K112∙(v11[−1 + i,−1 + j] − v11[−1 + i,1 + j] − v11[1 + i,−1 + j] + v11[1 + i,1 + j]))/(2∙hx1∙hy1) + (K112∙(−v11[i,−1 + j] + v11[i,1 + j])∙(v11[−1 + i,−1 + j] − v11[−1 + i,1 + j] − v11[1 + i,−1 + j] + v11[1 + i,1 + j]))/(4∙hx1∙Power(hy1,2)) + (K111∙(−w11[−1 + i,j] + w11[1 + i,j])∙(w11[−1 + i,j] − 2∙w11[i,j] + w11[1 + i,j]))/Power(hx1,3) + (K112∙(−w11[i,−1 + j] + w11[i,1 + j])∙(w11[−1 + i,−1 + j] − w11[−1 + i,1 + j] − w11[1 + i,−1 + j] + w11[1 + i,1 + j]))/(4∙hx1∙Power(hy1,2)))/2	

- Second equation:

(K133∙(u11[−1 + i,−1 + j] − u11[−1 + i,1 + j] − u11[1 + i,−1 + j] + u11[1 + i,1 + j]))/(4∙hx1∙hy1) +(K133∙(v11[−1 + i,j] − 2∙v11[i,j] + v11[1 + i,j]))/Power(hx1,2) + (K133∙(−w11[i,−1 + j] + w11[i,1 + j]) ∙(w11[−1 + i,j] − 2∙w11[i,j] + w11[1 + i,j]))/(2∙Power(hx1,2)∙hy1) + ((v11[i,−1 + j] − 2∙v11[i,j] + v11[i,1 + j])∙((K121∙(−u11[−1 + i,j] + u11[1 + i,j]))/hx1 + (K122∙(−v11[i,1 + j] + v11[i,1 + j]))/hy1 + (K122∙Power(−v11[i,−1 + j] + v11[i,1 + j],2))/(4∙Power(hy1,2)) + (K122∙Power(−w11[i,−1 + j] + w11[i,1 + j],2))/(4∙Power(hy1,2)) + (K121∙Power(−w11[−1 + i,j] + w11[1 + i,j],2))/(4∙Power(hx1,2))))/(2∙Power(hy1,2)) +(K133∙ (−w11[−1 + i,j] + w11[1 + i,j])∙(w11[−1 + i,−1 + j] − w11[−1 + i,1 + j] − w11[1 + i,−1 + j] + w11[1 + i,1 + j]))/(8∙Power(hx1,2) ∙hy1) + ((K121∙ (u11[−1 + i,−1 + j] − u11[−1 + i,1 + j] − u11[1 + i,−1 + j] + u11[1 + i,1 + j]))/(2∙hx1∙hy1) + (2∙K122∙ (v11[i,−1 + j] − 2∙v11[i,j] + v11[i,1 + j]))/Power(hy1,2) + (K122∙(−v11[i,−1 + j] + v11[i,1 + j]) ∙ (v11[i,−1 + j] − 2∙v11[i,j] ++ v11[i,1 + j]))/Power(hy1,3) + (K122∙(−w11[i,−1 + j] + w11[i,1 + j]) ∙ (w11[i,−1 + j] − 2∙w11[i,j] + w11[i,1 + j]))/Power(hy1,3) + (K121∙(−w11[−1 + i,j] + w11[1 + i,j]) ∙ (w11[−1 + i,−1 + j] − w11[−1 + i,1 + j] − w11[1 + i,−1 + j] + w11[1 + i,1 + j]))/(4∙Power(hx1,2) ∙hy1))/2. + ((−v11[i,−1 + j] + v11[i,1 + j])∙((K121∙ (u11[−1 + i,−1 + j] − u11[−1 + i,1 + j] − u11[1 + i,−1 + j] + u11[1 + i,1 + j]))/(2∙hx1∙hy1) + (2∙K122∙(v11[i,−1 + j] − 2∙v11[i,j] + v11[i,1 + j]))/Power(hy1,2) + (K122∙(−v11[i,−1 + j] + v11[i,1 + j])∙(v11[i,−1 + j] − 2∙v11[i,j] + v11[i,1 + j]))/Power(hy1,3) + (K122∙(−w11[i,−1 + j] + w11[i,1 + j])∙(w11[i,−1 + j] − 2∙w11[i,j] + w11[i,1 + j]))/Power(hy1,3) + (K121∙(−w11[−1 + i,j] + w11[1 + i,j])∙(w11[−1 + i,−1 + j] − w11[−1 + i,1 + j] − w11[1 + i,−1 + j] + w11[1 + i,1 + j]))/(4∙Power(hx1,2)∙hy1)))/(4∙hy1)	(A5)

- Third equation:

−(L122∙(w11[i,−2 + j] − 4∙w11[i,−1 + j] + 6∙w11[i,j] − 4∙w11[i,1 + j] + w11[i,2 + j]))/Power(hy1,4) + ((w11[−1 + i,j] − 2∙w11[i,j] + w11[1 + i,j])∙(−2∙Nx10[j] + (K111∙(−u11[−1 + i,j] + u11[1 + i,j]))/hx1 + (K112∙(−v11[i,−1 + j] + v11[i,1 + j]))/hy1 + (K112∙Power(−v11[i,−1 + j] + v11[i,1 + j],2))/(4∙Power(hy1,2)) + (K112∙Power(−w11[i,−1 + j] + w11[i,1 + j],2))/(4∙Power(hy1,2)) + (K111∙Power(−w11[−1 + i,j] + w11[1 + i,j],2))/(4∙Power(hx1,2))))/(2∙Power(hx1,2)) + ((w11[i,−1 + j] − 2∙w11[i,j] + w11[i,1 + j])∙((K121∙(−u11[−1 + i,j] + u11[1 + i,j]))/hx1 + (K122∙(−v11[i,−1 + j] + v11[i,1 + j]))/hy1 + (K122∙Power(−v11[i,−1 + j] + v11[i,1 + j],2))/(4∙Power(hy1,2)) + (K122∙Power(−w11[i,−1 + j] + w11[i,1 + j],2))/(4∙Power(hy1,2)) + (K121∙Power(−w11[−1 + i,j] + w11[1 + i,j],2))/(4∙Power(hx1,2))))/(2∙Power(hy1,2)) + (((K133∙(−u11[i,−1 + j] + u11[i,1 + j]))/(2∙hy1) + (K133∙(−v11[−1 + i,j] + v11[1 + i,j]))/(2∙hx1) + (K133∙(−w11[i,−1 + j] + w11[i,1 + j])∙(−w11[−1 + i,j] + w11[1 + i,j]))/(4∙hx1∙hy1))∙(w11[−1 + i,−1 + j] − w11[−1 + i,1 + j] − w11[1 + i,−1 + j] + w11[1 + i,1 + j]))/(2∙hx1∙hy1) + znak1∙(L112∙(w11[−1 + i,−1 + j] + w11[−1 + i,1 + j] + 4∙w11[i,j] + w11[1 + i,−1 + j] − 2∙(w11[−1 + i,j] + w11[i,−1 + j] + w11[i,1 + j] + w11[1 + i,j]) + w11[1 + i,1 + j]))/(Power(hx1,2)∙Power(hy1,2)) + znak1∙(L121∙(w11[−1 + i,−1 + j] + w11[−1 + i,1 + j] + 4∙w11[i,j] + w11[1 + i,−1 + j] − 2∙(w11[−1 + i,j] + w11[i,−1 + j] + w11[i,1 + j] + w11[1 + i,j]) + w11[1 + i,1 + j]))/(Power(hx1,2)∙Power(hy1,2)) −(4∙L133∙(w11[−1 + i,−1 + j] + w11[−1 + i,1 + j] + 4∙w11[i,j] + w11[1 + i,−1 + j] − 2∙(w11[−1 + i,j] + w11[i,−1 + j] + w11[i,1 + j] + w11[1 + i,j]) + w11[1 + i,1 + j]))/(Power(hx1,2)∙Power(hy1,2)) + ((−w11[−1 + i,j] + w11[1 + i,j])∙((2∙K111∙(u11[−1 + i,j] − 2∙u11[i,j] + u11[1 + i,j]))/Power(hx1,2) + (K112∙(v11[−1 + i,−1 + j] − v11[−1 + i,1 + j] − v11[1 + i,−1 + j] + v11[1 + i,1 + j]))/(2∙hx1∙hy1) + (K112∙(−v11[i,−1 + j] + v11[i,1 + j])∙(v11[−1 + i,−1 + j] − v11[−1 + i,1 + j] − v11[1 + i,−1 + j] + v11[1 + i,1 + j]))/(4∙hx1∙Power(hy1,2)) + (K111∙(−w11[−1 + i,j] + w11[1 + i,j])∙(w11[−1 + i,j] − 2∙w11[i,j] + w11[1 + i,j]))/Power(hx1,3) + (K112∙(−w11[i,−1 + j] + w11[i,1 + j])∙(w11[−1 + i,−1 + j] − w11[−1 + i,1 + j] − w11[1 + i,−1 + j] + w11[1 + i,1 + j]))/(4.∙hx1∙Power(hy1,2))))/(4.∙hx1) + ((−w11[−1 + i,j] + w11[1 + i,j])∙((K133∙(u11[i,−1 + j] − 2∙u11[i,j] + u11[i,1 + j]))/Power(hy1,2) + (K133∙(v11[−1 + i,−1 + j] − v11[−1 + i,1 + j] − v11[1 + i,−1 + j] + v11[1 + i,1 + j]))/(4∙hx1∙hy1) + (K133∙(w11[i,−1 + j] − 2∙w11[i,j] + w11[i,1 + j])∙(−w11[−1 + i,j] + w11[1 + i,j]))/(2.∙hx1∙Power(hy1,2)) + (K133∙(−w11[i,−1 + j] + w11[i,1 + j])∙(w11[−1 + i,−1 + j] − w11[−1 + i,1 + j] − w11[1 + i,−1 + j] + w11[1 + i,1 + j]))/(8∙hx1∙Power(hy1,2))))/(2∙hx1) + ((−w11[i,−1 + j] + w11[i,1 + j])∙((K121∙(u11[−1 + i,−1 + j] − u11[−1 + i,1 + j] − u11[1 + i,−1 + j] + u11[1 + i,1 + j]))/(2∙hx1∙hy1) + (2∙K122∙(v11[i,−1 + j] − 2∙v11[i,j] + v11[i,1 + j]))/Power(hy1,2) + (K122∙(−v11[i,−1 + j] + v11[i,1 + j])∙(v11[i,−1 + j] − 2∙v11[i,j] + v11[i,1 + j]))/Power(hy1,3) + (K122∙(−w11[i,−1 + j] + w11[i,1 + j])∙(w11[i,−1 + j] − 2∙w11[i,j] + w11[i,1 + j]))/Power(hy1,3) + (K121∙(−w11[−1 + i,j] + w11[1 + i,j])∙(w11[−1 + i,−1 + j] − w11[−1 + i,1 + j] − w11[1 + i,−1 + j] + w11[1 + i,1 + j]))/(4∙Power(hx1,2)∙hy1)))/(4∙hy1) + ((−w11[i,−1 + j] + w11[i,1 + j])∙((K133∙(u11[−1 + i,−1 + j] − u11[−1 + i,1 + j] − u11[1 + i,−1 + j] + u11[1 + i,1 + j]))/(4∙hx1∙hy1) + (K133∙(v11[−1 + i,j] − 2∙v11[i,j] + v11[1 + i,j]))/Power(hx1,2) + (K133∙(−w11[i,−1 + j] + w11[i,1 + j])∙(w11[−1 + i,j] − 2∙w11[i,j] + w11[1 + i,j]))/(2.∙Power(hx1,2)∙hy1) + (K133∙(−w11[−1 + i,j] + w11[1 + i,j])∙(w11[−1 + i,−1 + j] − w11[−1 + i,1 + j] − w11[1 + i,−1 + j] + w11[1 + i,1 + j]))/(8∙Power(hx1,2)∙hy1)))/(2∙hy1) −(L111∙(w11[−2 + i,j] − 4∙w11[−1 + i,j] + 6∙w11[i,j] − 4∙w11[1 + i,j] + w11[2 + i,j]))/Power(hx1,4)	(A6)

Power(x,n) = x^n^.

## Appendix C

The algorithm of calculations using a computer programme to solve the buckling and post-buckling problem is presented below in Figure A1.

## Appendix D

Internal pre-buckling forces for walls 1–5 of the member are shown below.

   Nx10(j) = Nx20(j) + ((b1 − hy1 j)(− Nx20(j) + Nx40(j)))/b3
Nx30(j) = Nx20(j) − j hy3/b3(Nx20(j) − Nx40(j))
   Nx50(j) = Nx40(j) − (hy1 j (− Nx20(j) + Nx40(j)))/b3
(A7)

where forces Nx20(j) and Nx40(j) are received from two boundary conditions (22):

(A8) $$\begin{array}{l} \sum \int_{0}^{b_{i}}N_{\mathrm{ix}}\mathrm{dy}=F\;(\mathrm{for}x=L)\\ \sum \int_{0}^{b_{i}}N_{\mathrm{ix}}y_{i}\mathrm{dy}=F\cdot e\;(\mathrm{for}x=L) \end{array}$$

y_i_—coordinate measured from major axis to edge of wall. Assuming a distribution of internal forces as a function of coordinate (y):

(A9) $$\begin{array}{l} \mathrm{Nx}30\left(y\right)=\mathrm{Nx}20-y/b3(\mathrm{Nx}20-\mathrm{Nx}40)\\ \mathrm{Nx}10(y)=\frac{\left(\mathrm{Nx}20-\mathrm{Nx}40\right)y}{b3}+\mathrm{Nx}20-b1/b3(\mathrm{Nx}20-\mathrm{Nx}40)\\ \mathrm{Nx}50\left(y\right)=\frac{\left(\mathrm{Nx}20-\mathrm{Nx}40\right)y}{b3}+\mathrm{Nx}40\\ \mathrm{Nx}40=\mathrm{factor}\cdot \mathrm{Nx}20 \end{array}$$

and taking two Equation (22), the formulae for Nx20 and ‘factor’ are obtained in the following form:

(A10) $$\begin{array}{l} \mathrm{Nx}20=\\ -\frac{(8{b1}^{3}-12{b1}^{2}b3+6b1b3(b3+2e)+b3(6b2(b3+2e)+b3(b3+6e)))F}{16{b1}^{4}+16{b1}^{3}(b2-b3)-24{b1}^{2}b2b3+8b1{b3}^{2}(3b2+b3)+{b3}^{2}(12{b2}^{2}+8b2b3+{b3}^{2})}\\ \mathrm{factor}=\frac{8{b1}^{3}-12{b1}^{2}b3+b3(b3(b3-6e1)+6b2(b3-2e1))+6b1b3(b3-2e1)}{8{b1}^{3}-12{b1}^{2}b3+6b1b3(b3+2e1)+b3(6b2(b3+2e1)+b3(b3+6e1))} \end{array}$$

where b1, b2, and b3 are the widths of the walls of investigated member.
